# Avoiding Void Holes and Collisions with Reliable and Interference-Aware Routing in Underwater WSNs †

**DOI:** 10.3390/s18093038

**Published:** 2018-09-11

**Authors:** Nadeem Javaid, Abdul Majid, Arshad Sher, Wazir Zada Khan, Mohammed Y. Aalsalem

**Affiliations:** 1 Department of Computer Science, COMSATS University Islamabad, Islamabad 44000, Pakistan; majidafridi28@gmail.com (A.M.); arshadsher92@gmail.com (A.S.); 2 Farasan Networking Research Laboratory, Department of Computer Science & Information System, University, Jazan 82822-6694, Saudi Arabia; wazirzadakhan@jazanu.edu.sa (W.Z.K.); aalsalem.m@jazanu.edu.sa (M.Y.A.)

**Keywords:** underwater wireless sensor networks, energy consumption, void hole, interference, backward transmission

## Abstract

Sparse node deployment and dynamic network topology in underwater wireless sensor networks (UWSNs) result in void hole problem. In this paper, we present two interference-aware routing protocols for UWSNs (Intar: interference-aware routing; and Re-Intar: reliable and interference-aware routing). In proposed protocols, we use sender based approach to avoid the void hole. The beauty of the proposed schemes is that they not only avoid void hole but also reduce the probability of collision. The proposed Re-Intar also uses one-hop backward transmission at the source node to further improve the packet delivery ratio of the network. Simulation results verify the effectiveness of the proposed schemes in terms of end-to-end delay, packet delivery ratio and energy consumption.

## 1. Introduction

To explore undersea available resources, UWSN is most feasible and effective solution. The aforesaid paradigm offers unique applications, for instance, inhabitant monitoring, tactical surveillance, disaster prevention, resource investigation, etc. [1,2,3]. UWSNs are different from the terrestrial ones in many aspects: (i) instead of radio signals, UWSNs use acoustic signals for communication; (ii) the topology of UWSNs is more dynamic than the terrestrial sensor networks; (iii) the deployment in underwater is relatively sparse; (iv) node localization in UWSNs is difficult as compared to terrestrial sensor networks [4,5,6,7]; and (v) the sensor nodes are energy limited and it is infeasible to replace or recharge their batteries after deployment [8]. The UWSNs face many challenges such as low bandwidth (<100 kHz), high propagation delay (1500 m/s) and high bit error rate.

Depth based routing [9,10,11,12,13] shows high packet drop due to void hole problem. Weighting depth and forwarding area division DBR (WDFAD-DBR) [14] only considers depth difference between two hops to avoid void hole. However, considering two hops does not eliminate void hole. The void hole is an area from where data packets cannot be delivered to the destination. This is because of the unavailability of nodes in the communication range of the source node. It could occur at the time of random deployment when nodes get placed far away from each other and are never able to communicate with the network nodes. Moreover, it could be due to displacement of nodes with water currents as well as because of high burden of data which suddenly depletes node battery.

To resolve the issue of void hole problem, various routing strategies are presented by the research community, e.g. WDFAD-DBR [14]. This algorithm assigns weights to neighbor nodes for effective forwarder selection and avoids immutable nomination of the forwarder node. Although it assigns weights to all neighbors and computes holding time to save battery, it considers only one selection parameter which is distance and obtains neighbor information up to two hops. This scheme gives more weight to distance parameter, which enables selection of the same forwarder until its death. Thus, the void hole is not avoided at all.

There is also a backward transmission mechanism which finds alternate paths to deliver data at the destination [15]. It traverse backward nodes, until a neighbor is available which can deliver the data successfully to the base station. However, there is a problem of communication overhead which degrades the network performance. Thus, a protocol is desired which can minimize the communication overhead to increase network lifetime. In this regard, a protocol which uses unique hop identifiers to minimize overhead and improves the network lifetime along with the void avoidance [16]. However, this scheme is not effective because in harsh acoustic environment, water currents are major influential factor which make it difficult to obtain hop id all the time.

Therefore, to avoid void hole, new routing protocol(s) needs to be proposed. In proposed routing protocols, we use sender based approach to avoid void hole and reduce the probability of collision by considering channel inference. Moreover, the proposed protocols also reduce duplicate packet transmission. It is important to note that this research work is extended form of the work in [17].

The rest of the paper is organized as follows. In Section 2, we discuss some well-known routing protocol with their drawbacks for UWSNs. Details of the proposed schemes are given in Section 3. Section 4 presents theoretical analysis. A detailed analysis about the performance evaluation of proposed protocols is given in Section 5. Finally, conclusions are drawn in Section 6.

## 2. Related Work

In this section, the existing related works are discussed in two categories; localization-aware and localization-free routing protocols.

### 2.1. Localization-Free Routing Protocols

Depth based routing (DBR) [9], energy-efficient DBR (EEDBR) [10], adaptive mobility of courier nodes in threshold-optimized depth based routing (AMCTD) [11], improved AMCTD (iAMCTD) [12] and delay-sensitive routing schemes [13] are popular localization-free routing protocols. In DBR, the neighbor with least depth is selected as forwarder. The holding time is computed based on depth of the forwarder node to avoid redundant transmissions at the destination. The node having smaller depth with respect to destination is always given the highest priority to proceed with the data delivery. In EEDBR, two control parameters are used to ensure cyclic selection of forwarder nodes. The parameters are: depth and energy. Based on these parameters, source node initiates a data packet within the communication range. Every node receives the packet and computes its holding time, then node with lowest holding time proceed with the data communication. AMCTD introduces the approach of courier nodes to achieve higher delivery ratio, better network stability and optimized lifetime. It uses weight function for selecting next forwarder of data packet on the basis of network density. Optimal weight computation not only balances load among the network nodes but also optimizes the holding time. These protocols do not find next hop eligible forwarder when void hole occurs, which results in low packet delivery ratio. Javaid et al. extended AMCTD protocol to iAMCTD protocol by considering three routing metrics for calculating weight function: signal quality index (SQI), energy cost function (ECF), and depth-dependent function (DDF). Moreover, it implements on-demand data routing and maximizes the lifetime of UWSNs by optimized mobility pattern of courier nodes. Javaid et al. [13] proposed three routing schemes: delay-sensitive DBR (DSDBR), delay-sensitive EEDBR (DSEEDBR), and delay-sensitive AMCTD (DSAMCTD). These protocols formulate delay-efficient priority factors (PF) and delay-sensitive holding time (DSH${}_{T}$) to minimize end-to-end delay. Ayaz et al. proposed hop-by-hop dynamic addressing based (H2-DAB) [16] beacon-based routing protocol, which does not require any location information. In H2-DAB, each sensor node is assigned a unique hop-id by using beacon message. A surface sink broadcasts a beacon message, while the receiving nodes are assigned hop-ids. The receiving nodes increment the hop-id and rebroadcast it. In this way, each node is assigned a unique hop-id. This hop-id is used as routing metric where sensor node selects next forwarder with the small hop-id. Wahid et al. proposed reliable energy efficient routing protocol (R-ERP${}^{2}$R) [18] to improve the reliability of the delivered data and efficiently utilize the node battery. The R-ERP${}^{2}$R uses multiple metrics: link quality, physical distance and residual energy to nominate next forwarder. In [19], Basagni et al. proposed channel aware routing protocol (CARP), which combines link quality and hop count as routing metrics, to avoid void hole effectively. It uses cross layer approach for channel access and relay selection. In CARP, node is selected as relay if it has a history of successful delivery to its neighbor node with high residual energy. E-CARP [20] is the enhanced version of CARP. It reduces energy consumption of the network by avoiding control packets during relay selection and reduces sensory data to sink, when the underwater environment is relatively steady. In [15], hydraulic pressure based anycast (HydroCast) routing scheme is proposed to avoid void hole using backward transmissions. This process continues until the neighbor node finds lower pressure node. The occurrence of void hole can be avoided through efficient energy consumption among the network nodes. In this regard, Wan et al. proposed cluster based algorithm to cater the problem of high data at the nodes deployed nearby the sink [21]. To overcome the issue of imbalanced data traffic, authors introduced radius competition strategy based on the residual energy and distance of the node from the sink. The cluster head is elected with highest energy among the neighbor nodes. Moreover, the number of neighbors purely depends on the distance from the sink because the smaller the distance is, higher will be the data traffic, thus smaller radius is picked to form a cluster for efficient energy consumption. Similarly, energy efficiency is achieved via discovering most reliable path in the network using the sink nodes deployed at the surface of the water [22]. This scheme advances the discovery of path by initiating the data packet from the source node to next hop node placed towards the destination. The process continues until surface is reached and gets helpful information from the surface node. Additionally, for an efficient node battery utilization, splice method is introduced to compare the energy consumption associated with each link. After comparison, the shortest path with minimal energy required to deliver data successfully is picked to resume the nodes communication.

### 2.2. Localization-Aware Routing Protocols

Jornet et al. in [23] avoided unnecessary data transmissions in the network by proposing focused beam routing (FBR) algorithm. The FBR uses R/CTS (ready/clear to send) based mechanism with adaptive power transmission level. When the network gets sparse, every source node makes several attempts to select forwarder node by broadcasting RTC packet. This mechanism dissipates surplus energy along with higher delay.

Fuzzy logic is used to calculate the desirableness factor to forward the data packets in acoustic networks [24]. The information of energy and position is utilized to compute the effectiveness of next hop node in fuzzy based forwarding protocol. The fuzzy logic system takes as input, the node battery level, trajectory and distance of the node from the sink node. The consideration of multiple parameters helped in achieving higher network lifetime and lower end-to-end delay.

To control the flooding of information, directional flooding-based routing (DFR) scheme is proposed in [25]. The earlier said scheme restricts the participation of forwarders between source to destination. The communication area of flooding is defined based on acquired link quality and angle between the transmitter and receiver nodes. The DFR saves significant amount of energy by using directional flooding. Further, the effective neighbor is difficult to elect as the sparsity of the network increases. In addition, it adjusts from the defined static power levels, which consume more battery, especially when the distance is high.

A localization algorithm is proposed in [26] to handle the uncertainty of the anchor nodes. The use of pressure sensors allowed the authors to find only two-dimensional coordinates. The work computed the uncertainty of the targeted node along with the issues of bending ray. The performance is measured via simulating against schemes with known anchor nodes location and locations calculated through straight line mechanism between two nodes. The errors of straight line is mapped via measuring time of flight and range errors are catered through multivariate gaussian distribution. The trajectory is calculated through gaussian beam tracing method. This scheme gained localization accuracy up to 49.4% against the traditional schemes.

In [27], a vector-based forwarding (VBF) scheme saves energy using pipeline mechanism. This restricts the node involvement; however, rigid forwarding range degrades the network lifetime. Nicolaou et al. extended VBF to HH-VBF (hop-by-hop VBF) [28] protocol. This algorithm makes forwarding range decision at each hop based on the neighbor information, which makes it suitable for sparse networks. However, due to constant network radius in HH-VBF, the load is distributed unevenly among the entire networks nodes. In [29], an adaptive hop-by-hop VBF (AHH-VBF) routing protocol is presented. In AHH-VBF, during packet transmission, the radius of pipeline is adaptively adjusted at each hop to reduce energy expenditure. Furthermore, the holding time of the packet is computed according to the distance between forwarder node and destination to reduce end-to-end delay. In AHH-VBF, increasing the radius of pipeline does not resolve the void hole problem.

The existing schemes: WDFAD-DBR [14], Hydrocast [15] and H2-DAB [16] avoid void hole problem using different approaches. The WDFAD-DBR uses neighbor information up to two hops to overcome void issue. The Hydrocast traverses backwards until it finds a suitable forwarder which can deliver the data packet. H2-DAB involves unique id mechanism to deliver the data to the sink node. These schemes take precautionary measures when void node occurs. However, our proposed work is different in terms of data forwarding mechanism. The complete route is discovered before delivering the data to neighbor node because information up to two hops never eliminates the void hole problem. If void problem occurs, we only move one hop in the backward direction to resume the greedy forwarding process. To minimize the interference, neighbor node with fewer neighbors is selected. We have updated neighbor tables using piggy back mechanism to reduce communication overhead. The detailed discussion of the proposed work is presented in the upcoming sections.

Detailed comparison of the routing protocols for UWSNs discussed above is given in Table 1.

## 3. Proposed Schemes

The propose schemes use a sender based approach for the selection of next forwarder node.

**Figure 1:**
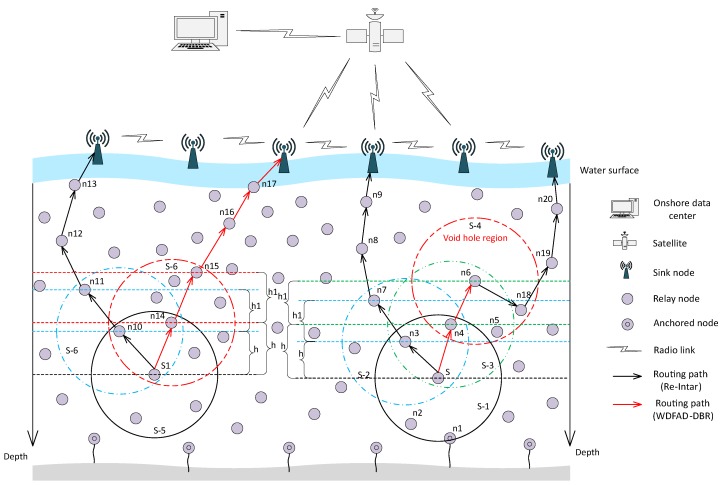
Network architecture of the proposed Re-Intar, illustrating void hole problem and collision avoidances.

**Network model:** In proposed schemes, we assume a multi-sink network architecture [30,31]. The network architecture is housed with numerous sensor nodes (anchored and normal nodes), as shown in Figure 1. As depicted in the network model (Figure 1), many sinks are placed on the water surface which are stationary and equipped with radio and acoustic modems. The radio modem is for communication on the land to deliver data to the base station. While, acoustic modem is to gather data from the nodes deployed inside the water. There are also anchored nodes at the bottom of water only provided with acoustic modem. Moreover, there many free floating nodes which are moving with water currents and we assume that each knows its location at the time of deployment. The assumption made is that data delivered from any acoustic node to any of the sink is considered to be available at all the sinks. Moreover, we have considered mobility of the nodes due to water currents in horizontal direction, while movement in vertical direction is small and thus negligible.

### 3.1. Proposed Scheme 1: Intar

Intar protocol consists of two phases: network setup phase and data forwarding phase. Details are given in the following subsections.

#### 3.1.1. Network Setup Phase

**Figure 2:**
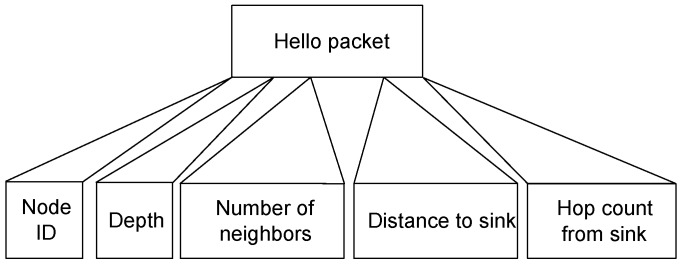
Hello packet format.

In the setup phase of Intar, every node broadcasts a message to find its neighbors and hop count, which is computed through beacon generated from the sink and Euclidean distance from the sink. This information is exchanged with neighbors of every node with the help of HELLO packet, as shown in Figure 2. In Intar, hello packet consists of four fields i.e., node ID, number of neighbors, distance to sink and hops from the sink, whereas, in Re- Intar, hello packet consists of one more field, i.e., depth.

When sensor node receives hello packet, it stores this information in the neighbor table. In Intar, neighbor table of each sensor node consists of four fields, i.e., NeighID, NumNeighbor, HopSink, and DistNeighbor. In addition, neighbor table of Re-Intar consists of two more fields, i.e., Depth and Timestamp. The neighbor table is illustrated in Table 2, where NeighID is the unique id (address) of a sensor node, NumNeighbor is the total neighbors available in the transmission range, HopSink denotes the hop-count using Euclidean distance from the sink, DistNeighbor is the Euclidean distance towards that neighbor, Depth denotes the depth difference with that neighbor, and Timestamp represents the time to update neighbor entry in the table. Furthermore, we also exploited piggy-backing mechanism to lower neighbor requests. When sender node sends data packet, it also includes hello packet information in the data packet. Upon receiving the data packet from sender node, receiver node updates the neighbor entry in the neighbor table if its depth is greater than the sender node.

#### 3.1.2. Data Forwarding Phase

The source/sender node of the data packet selects the next forwarder from its PFNs on the bases of cost function (CF) value, which is calculated as follows:

(1) $$CF\left(j\right)=\frac{Dist(i,j)}{Hop(j)\times Neighbor(j)},$$

where Hop(j) is the number of *j*th PFN from sink, Neighbor(j) is the number of neighbors of *j*th PFN and Dist(i,j) is the distance between *j*th PFN and source node *i*. According to Equation (1), PFN having least number of neighbors, minimum number of hops from the sink and maximum distance from the sender node have the maximum CF value. As WDFAD-DBR only considers depth difference between two hops to avoid void hole. However, considering two hops does not eliminate void hole, as shown in Figure 1. Moreover, it does not consider channel interference, thus collision probability is high because the packet is delivered to sink through high node density region. We reduce collision probability and avoid void hole by selecting a PFN having path to sink with least number of neighbors as the next forwarder of the packet from S/S1 to sink, as shown in Figure 1.

Source node selects PFN with maximum CF value, includes its ID in the data packet and broadcasts it to its neighbor nodes. Upon receiving the data packet, every node compares its ID with the ID which is received in the data packet. If the received ID matches, neighbor node accepts a data packet from the source/sender node and is selected as next forwarder/sender of the data packet. All other neighbor nodes discard the data packet. This process continues until data packet reaches the sink node.

### 3.2. Proposed Scheme 2: Re-Intar

Re-Intar is proposed to improve the packet delivery ratio and reduces end-to-end delay of the network. It addresses the limitations of both WDFAD-DBR and Intar techniques. Similar to Intar, Re-Intar protocol also consists of two phases: network setup phase and data forwarding phase. Re-Intar differs from Intar only in the data forwarding phase, which is discussed in the upcoming subsection.

#### Data Forwarding Phase

The CF value of PFN in Re-Intar depends on four parameters:

(2) $$CF\left(j\right)=\frac{Dist\left(i,j\right)\times dth_{diff}\left(i,j\right)}{Hop(j)\times Neighbor(j)}.$$

where $dth_{diff}\left(i,j\right)$ is the depth difference between source node *i* and PFN *j*. The remaining parameters are similar to Equation (1). In Re-Intar, when a source/sender node generates a data packet, it uses Equation (2) and selects PFN having maximum value of CF as next forwarder. This process continues until the data packet is delivered to sink.

To improve packet delivery ratio of Intar, Re-Intar also uses one hop backward transmission in the case of void region, a source node looks for non-PFNs in its transmission range and chooses one-hop backward node for data forwarding. When a source node generates data packet, it looks for PFN in its transmission range. If PFN is not found, then it looks for non-PFN in its transmission range and selects the one with minimum distance to sink as next forwarder of data packet. Algorithm 1 shows the procedure for data forwarding in Re-Intar.
**Algorithm 1** Data forwarding algorithm.1:**procedure**Next–forwarder selection2:    *N* ← Total number of node in network3:    Tx−range ← Transmission range4:    PFN ← Potential forwarder node having depth less than Source/sender node5:    Non−PFN ← Node having depth greater than Source/sender node6:    **for** Each node *i* ∈ *N* **do**7:        Node(i) ← Generate data packet8:        **if** SinkinTx−Range **then**9:           Packet to sink10:           Goto step 6:11:        **else**12:           **if** PFNexist **then**13:               Nextforwarder ← Select PFN using Equation (2)14:               Go to step 8:15:           **else**16:               **if** Non−PFNexist **then**17:                   Nextforwarder ← Select non-PFN with minimum distance to sink18:                   Go to step 8:19:               **else**20:                   Packet dropped21:                   Go to step 6:22:               **end if**23:           **end if**24:        **end if**25:    **end for**26:**end procedure**

**Figure 3:**
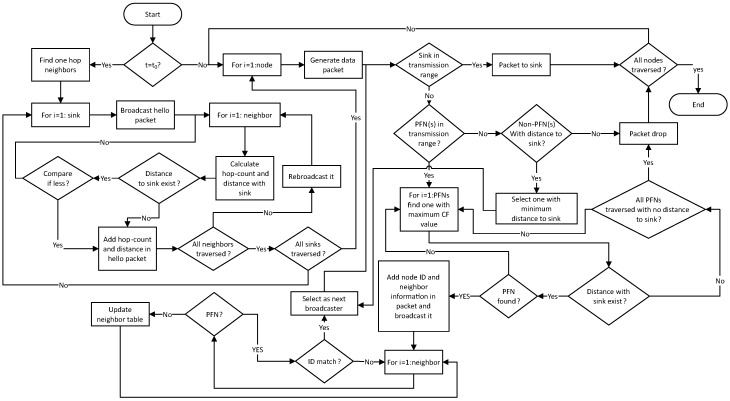
The Re-intar: flow chart.

When a node sends data packet to next forwarder node, the data packet may not reach the next forwarder node due to bad channel condition [30]. To handle such situation, the sender node buffers the data packet and waits for certain time to overhear it from the receiver node. On overhearing the data packet from receiver node, the sender node removes the packet from its buffer. If the data packet is not heard within the waiting time, the sender node selects next forwarder from its PFNs with second highest value of CF, if other PFN exists. Otherwise, it selects the first one and rebroadcasts the packet. The waiting time depends on the propagation distance between the sender and receiver. The flow chart of Re-intar protocol is shown in Figure 3.

## 4. Theoretical Analysis

This section describes the theoretical analysis of the proposed Re-Intar protocol. In Re-Intar, the further is the node from the sink node, the more hops the packet needs to reach the sink node.

### 4.1. Packet Delivery Probability Estimation

The acoustic channel attenuation over a distance *d* for a signal of frequency *f* due to Rayleigh fading is expressed as:

(3) $$A\left(d,f\right)=d^{k}a{\left(f\right)}^{d},$$

where a(f) is the absorption coefficient and *k* is the spreading factor which defines the geometry of spreading: k=2 for spherical spreading, k=1 for cylindrical spreading and k=1.5 for practical spreading [3]. The absorption coefficient a(f) (in dB/km) for *f* (in kHz) is calculated using Thorps formula [32] given by:

(4) $$10\log a\left(f\right)=\frac{0.11\times f^{2}}{1+f^{2}}+\frac{44\times f^{2}}{4100+f}+2.75\times 10^{-4}f^{2}+0.003.$$

The average signal to noise ratio (SNR) over *d* is given as follows:

(5) $$\Gamma \left(d\right)=\frac{E_{b}/A\left(d,f\right)}{N_{0}}=\frac{E_{b}}{N_{0}d^{k}a{\left(f\right)}^{d}},$$

where $E_{b}$ represents energy per bit and $N_{0}$ represents noise power spectral density in an additive white gaussian noise (AWGN) channel. we use binary phase shift keying (BPSK), for which the probability of error over *d* is calculated as follows [33]:

(6) $$p_{e}\left(d\right)=\frac{1}{2}\left(1-\sqrt{\frac{\Gamma (d)}{1+\Gamma (d)}}\right).$$

The delivery probability of a packet of *m* bits over distance *d* between any pair of nodes is given as follows:

(7) $$p\left(d,m\right)={\left(1-p_{e}\left(d\right)\right)}^{m}.$$

Throughout in this paper, we shortly abbreviate the above notation as $p\left(h_{\left(j\right)}\right)$.

### 4.2. Packet Delivery Ratio Analysis

Let the total number of generated packets at each node be λ. Then, the throughput of the network is expressed as:

(8) $$Th=\sum_{i=1}^{n}\left(\prod_{j=1}^{h(i)}p\left(h_{\left(j\right)}\right)\right)\lambda .$$

where *n* is the number of nodes, h(i) is the number of hops of *i*th node and $p\left(h_{\left(j\right)}\right)$ is the probability of packet delivery at the *j*th hop. For n nodes in the network, nλ are the total number of generated packets in the network. The packet delivery ratio of the network can be expressed as:

(9) $$PDR=Th/n\lambda =\frac{1}{n}\sum_{i=1}^{n}\left(\prod_{j=1}^{h(i)}p\left(h_{\left(j\right)}\right)\right).$$

### 4.3. Average End-to-End Delay Analysis

The total end-to-end delay for λ generated packets in the network with contending channel can be computed as follows:

(10) $$D_{T}=\sum_{i=1}^{n}\left(\prod_{j=1}^{h(i)}p\left(h_{\left(j\right)}\right)\right)\lambda h\left(i\right)\left(t_{s}+t_{p}+t_{r}\right).$$

where $t_{s}$ is the delay for sending/forwarding a packet, $t_{r}$ is the delay for receiving a packet and $t_{p}$ is the propagation delay. The propagation delay for any pair of nodes with a distance *d* is given as $t_{p}\left(d\right)=d/c$, where c is the speed of acoustic signal in water. The average end-to-end delay per packet in the entire network is given as follows:

(11) $$\bar{D}=\frac{D_{T}}{Th}=\frac{\sum_{i=1}^{n}\left(\prod_{j=1}^{h(i)}p\left(h_{\left(j\right)}\right)\times h\left(i\right)\left(t_{s}+t_{p}+t_{r}\right)\right)\lambda }{\sum_{i=1}^{n}\left(\prod_{j=1}^{h(i)}p\left(h_{\left(j\right)}\right)\right)\lambda }.$$

### 4.4. Average Energy Analysis

The total energy consumption of the network in one data gathering round without contention is given as:

(12) $$E_{r}=\sum_{i=1}^{n}\left(e_{g}+\sum_{j=1}^{h(i)}\left(e_{t}+N_{j}\times e_{r}\right)\right).$$

where Nj is the number of neighbor nodes at the $j_{th}$ hop, $e_{g}$ is the average energy required to generate one data packet, $e_{t}$ is the transmitting energy, i.e., the average energy required to transmit one data packet from source node to destination/relay node, and $e_{r}$ is the receiving energy, i.e., the average energy required to receive one data packet from the source node. The total energy consumption for λ generated packets in the network with contention can be computed using Equations (8) and (12) as:

(13) $$E_{t}=\sum_{i=1}^{n}\left(e_{g}+\prod_{j=1}^{h(i)}p\left(h_{\left(j\right)}\right)\sum_{j=1}^{h(i)}\left(e_{t}+N_{j}*e_{r}\right)\right)\lambda .$$

The average energy consumption per successful packet in the entire network is given as follows:

(14) $$\bar{E}=\frac{\sum_{i=1}^{n}\left(e_{g}+\prod_{j=1}^{h(i)}p\left(h_{\left(j\right)}\right)\sum_{j=1}^{h(i)}\left(e_{t}+N_{j}*e_{r}\right)\right)\lambda }{\sum_{i=1}^{n}\left(\prod_{j=1}^{h(i)}p\left(h_{\left(j\right)}\right)\right)\lambda }.$$

## 5. Performance Evaluation

For performance evaluation, we compared Re-Intar and Intar with three existing schemes: WDFAD-DBR, Hydrocast and H2-DAB. The existing schemes avoid void hole in both sparse and dense network deployments. We compared the existing work by increasing the node density to show the effectiveness of the proposed work. The comparison was made on: packet delivery ratio, energy tax, end-to-end delay and accumulative propagation distance. In simulations, we randomly deployed 100–500 sensor nodes in a three-dimensional region of 10 km × 10 km × 10 km. Nine sinks were uniformly placed at the surface of the water. We assumed that the sinks remain stationary after deployment and sensor nodes change their position with water current. It is important to note that, each sensor node moved in a horizontal two-dimensional direction, i.e., X-Y plane (random walk 2D mobility model). The movement speed of a node was 1–3 m/s. In vertical direction, the movement of node was usually small, thus negligible [34,35]. The simulation parameters are listed in Table 3.

To evaluate the performance of Intar and Re-Intar, we used the metrics enlisted in Table 4.

**Figure 4:**
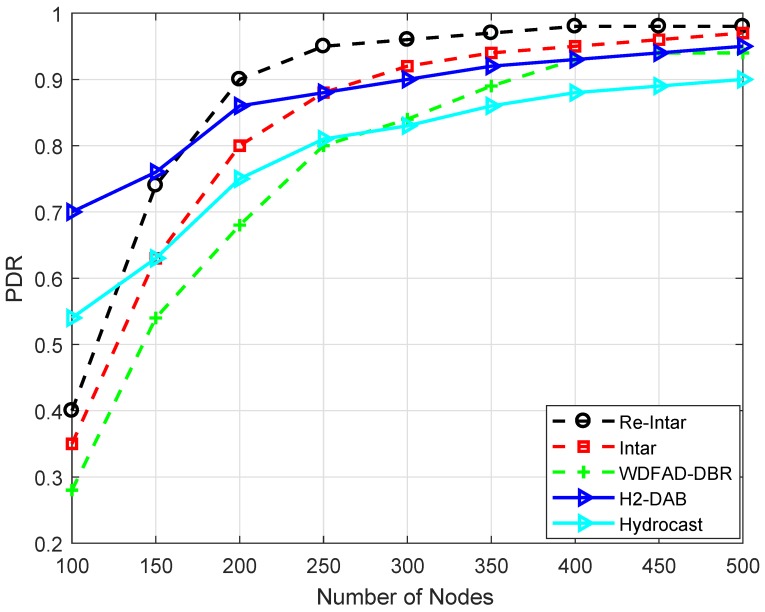
PDR of the existing and proposed protocols with varying number of nodes.

Figure 4 shows that PDR of WDFAD-DBR, Intar and Re-Intar increases with increase in the number of nodes. When the network is sparse, the probability of void hole is high and fewer packets are successfully delivered to the destination. In dense network, more packets are successfully delivered to the destination. Figure 4 also shows that the performance of Re-Intar protocol is better than WDFAD-DBR and Intar in terms of PDR in both sparse and dense networks. In WDFAD-DBR, PFN considers the depth of current hop and the depth of next expected hop while forwarding packet from the source node. Two-hop forwarding metric does not eliminate the chances of void hole occurrence, which decreases its PDR. Intar protocol shows better PDR than WDFAD-DBR because it avoids void hole(s) due to consideration of end-to-end path. In addition to the consideration of end-to-end path, Re-Intar also uses one backward transmission resulting in further improvement in terms of PDR. Moreover, WDFAD-DBR has no mechanism to reduce interference in high node densities. Intar and Re-Intar reduce channel interference by selecting PFN with the fewest neighbors as the next forwarder. Thus, the performance of Intar and Re-Intar is better than WDFAD-DBR when node density is increased. Relatively, Re-Intar is approximately 16.96% more efficient than WDFAD-DBR and 7.26% more efficient than Intar in PDR.

Hyrocast has lower PDR than all the proposed and existing (H2-DAB) schemes. The reason is the backward transmissions which increase the path length and result in higher energy dissipation. The more energy dissipation means lower lifetime along with the smaller PDR which is evident from the Figure 4. The H2-DAB performs better initially, but, as the node density increases, the broadcast of hop id message to find unique identifiers results in interference. When node number is 100, the PDR is 70%, while it ends up with almost the same PDR as WDFAD-DBR.

**Figure 5:**
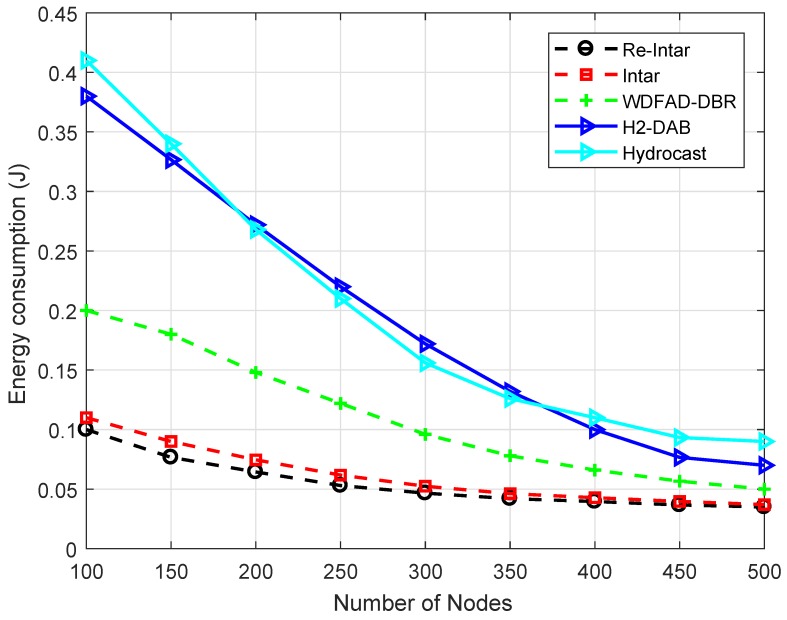
Energy tax of the existing and proposed protocols with varying number of nodes.

The energy consumption of H2-DAB is almost 35% higher at 100 node density as compared to proposed schemes. However, it decreases gradually with the increase in node number from 150 to 300, where the difference approximately is 7%. This difference remained until node density 500. The case with Hydrocast is similar, where the gap is around 40% at 100 node number. The sudden decrease can be observed in the Figure 5. This ends with the gap of 9% from proposed techniques and almost 3% of difference from H2-DAB.

**Figure 6:**
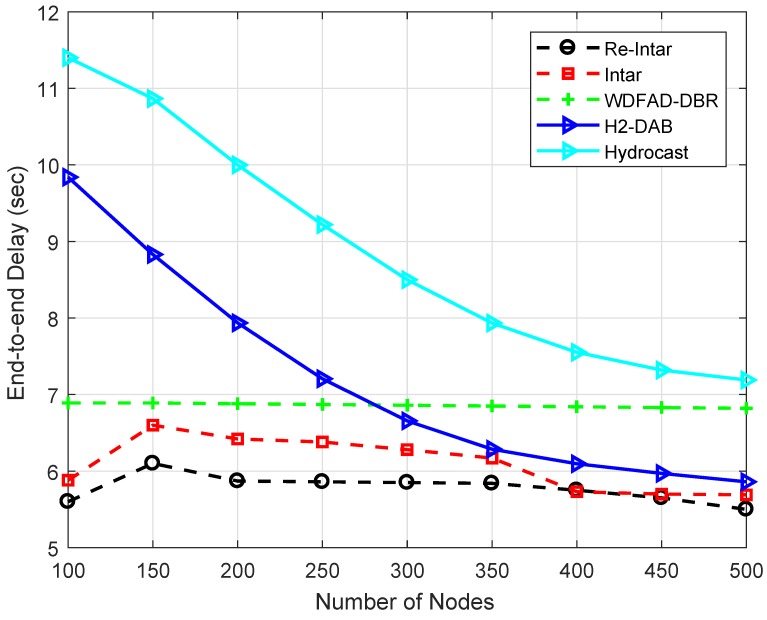
End-to-end delay of the existing and proposed protocols with varying number of nodes.

Figure 6 shows that the end-to-end delay of all the three protocols first increases with increase in the number of nodes but then decreases. When the network is too sparse, most of the packets are delivered from low depth nodes. The end-to-end delay increases with increasing number of nodes because the probability of void hole decreases and the probability of path establishment increases from the high depth nodes, which increases the end-to-end delay per packet. When number of nodes exceed 200, end-to-end delay decreases due to more PFNs in a dense network which reduces the number of hops between source and sink. WDFAD-DBR has high end-to-end delay due to packet holding time. In contrast, Re-Intar and Intar do not consider holding of the packet. Thus, end-to-end delay is reduced only to propagation delay, transmitting delay and receiving delay. In Intar, high end-to-end delay is due to long propagation path because of avoiding channel interference. Re-Intar reduces propagation distance of the packet by using depth of PFN. However, Intar shows better performance than Re-Intar when node number is 100 in network because Re-Intar also uses backward transmission. Relatively, Re-Intar is approximately 37.16% more efficient than WDFAD-DBR and 10.21% more efficient than Intar in end-to-end delay.

It can be observed that delay in both Hydrocast and H2-DAB is higher than the proposed schemes. Hydrocast has more delay as we have mentioned in the discussion of PDR that it prefers backward transmissions to handle void hole problem. In this process, it has to traverse more hops which increase delay and also dissipate more energy as well. H2-DAB has more delay, but, at the end, it outperforms both WDFAD-DBR and Hydrocast. This scheme uses unique identifiers which reduce the path length with the increase in node density.

**Figure 7:**
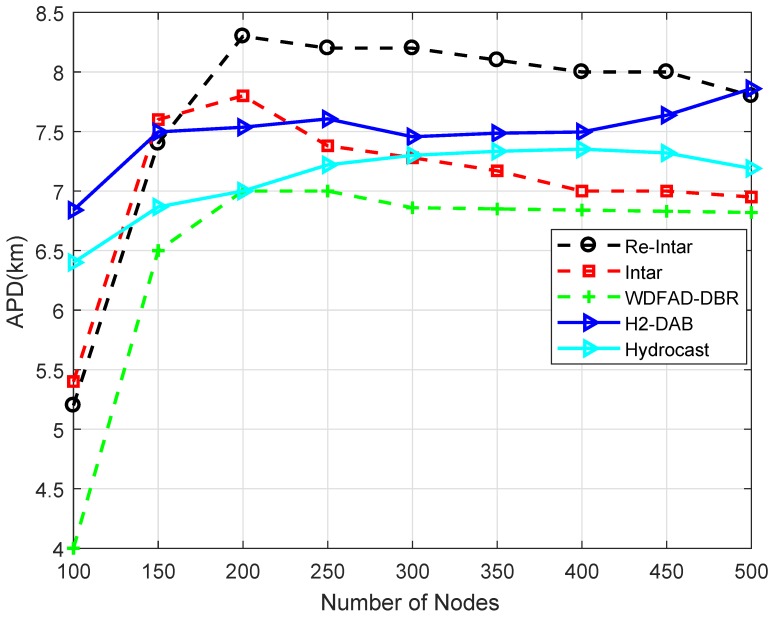
APD of the existing and proposed protocols with varying number of nodes.

Similar to end-to-end delay, APD of all the three protocols first increases with increase in the number of nodes and then decreases, as shown in Figure 7. When the network is too sparse, most of the packets are delivered from low depth nodes. When the number of node increases, high depth nodes communicate with sink which increases APD per delivered packet. When the number of nodes exceed 200, APD decreases because the probability of selecting a PFN with minimum distance to sink increases which reduces APD per delivered packet. Figure 7 shows that the performance of WDFAD-DBR is better than Intar and Re-Intar because it selects a PFN with least depth as next forwarder. These two protocols bypass the shortest path due to avoidance of channel interference which increases their APD. Re-Intar shows better performance than Intar because Re-Intar reduces propagation distance of the packet by using depth of PFN. Relatively, Re-Intar is approximately 8.82% more efficient than Intar and 8.36% less efficient than WDFAD-DBR in APD.

The APD of both Hydrocast and H2-DAB schemes is higher than Intar. Re-Intar has higher APD than existing schemes from node number 200 to 450, while it shows slightly lower APD than H2-DAB at 500 node number. The Hyrocast outperforms Re-Intar in APD throughout the network operations but still lacks the effectiveness as compared to Intar scheme. This is because in proposed work only one backward hop is traversed, whereas Hydrocast traverses back until the eligible forwarder is nominated to resume the network operations.

### Performance Trade-Offs

The performance trade-offs of Re-Intar with selected protocols are listed in Table 5. WDFAD-DBR makes a routing decision according to two metrics: depth of current hop and depth of next expected forwarding node, to avoid void hole. However, considering these two metrics cannot eliminate void hole. Therefore, WDFAD-DBR shows low PDR, as shown in Figure 4, and high energy consumption due to high packet drop in the sparse network (Figure 5). Moreover, it selects a PFN with least depth as next forwarder, achieves low APD, as shown in Figure 7, and less energy consumption in dense network, as shown in Figure 5, but, due to holding time, shows high end-to-end delay, as shown in Figure 6. Intar protocol does not consider holding time of the packet and avoids void hole by following end-to-end path from source node to sink. Therefore, Intar protocol shows high PDR (Figure 4), less energy consumption in the sparse network (Figure 5) and low end-to-end delay (Figure 6) compared to WDFAD-DBR at the cost of high APD (Figure 7) and high energy consumption (Figure 7). Besides end-to-end path Re-Intar also uses one-hop backward transmission and reduces APD of the Intar resulting in high PDR, as shown in Figure 4, low end-to-end delay (Figure 6) and less energy consumption in the sparse network (Figure 5) at the cost of high APD than WDFAD-DBR, as shown in Figure 7.

## 6. Conclusions

In this article, we have presented reliable and interference-aware routing protocols for UWSNs. In the proposed work, selection of PFN with already free established path to sink successfully avoided the void hole. By using sender based approach, the proposed protocols successfully avoided duplicate packet transmission. Moreover, selection of PFN with least number of neighbors reduced the probability of collision in dense networks. One hop backward transmission in Re-Intar shows improvement over WDFAD-DBR and Intar in terms of PDR. Moreover, using depth of PFN reduced APD, end-to-end delay and energy consumption as compared to Intar. Simulation results verify the effectiveness of proposed schemes in terms of PDR, end-to-end delay and energy consumption.

## Figures and Tables

**Table 1:** Comparison of different existing schemes.

Protocol	Features	Flaws/Deficiency	Advantages Achieved
DBR [9]	Localization-free DBR protocol for underwater monitoring that handles dynamic networks.	Duplicate packets transmission, excessive energy consumptions and high end-to-end delay because of holding time. Due to greedy approach, void hole occurs. Inefficient for sparse and highly dense networks.	Improved network lifetime and data delivery ratio.
EEDBR [10]	Localization-free routing protocol for underwater monitoring and surveillance applications with controlled flooding.	High end-to-end delay due to holding time, no mechanism to avoid void hole and high energy consumption in dense networks.	Improved network lifetime, data delivery ratio and minimized energy consumption.
AMCTD [11]	Localization-free routing protocol with adaptive mobility of courier nodes.	High transmission loss due to distant transmissions of medium-depth nodes. Inefficient for data-sensitive applications due to mobility of courier nodes especially during instability period.	Prolonged network lifetime and reduced energy expenditure of low-depth sensor nodes specifically in stability period. Upholds the network throughput in the sparse condition with adaptive mobility of courier nodes.
iAMCTD [12]	Localization-free routing for time critical applications along with adaptive mobility of courier nodes.	Overhead in terms of control packets exchange and the problem of encounters void hole exist.	Prolonged network lifetime and reduced transmission loss. Minimized end-to-end delay and critical data loss in delay-sensitive applications.
Delay-sensitive schemes [13]	Delay-sensitive routing protocols as an improvement to localization-free routing schemes; DBR, EEDBR, and AMCTD.	Duplicate packets transmission in DSDBR, high energy consumption in dense networks in DSEEDBR and high transmission loss due to distant transmissions of medium-depth nodes in DSAMCTD.	Minimized total energy consumption, transmission loss and average end-to-end delay.
H2-DAB [16]	Localization-free beacon based routing scheme for critical underwater monitoring missions, route selection is based on hop-id.	Request and replay inquiry act as overhead and increased end-to-end delay as well as energy consumption of the network.	No need for full dimension location information. Achieved high data delivery ratio in both sparse and dense network.
R-ERP${}^{2}$R [18]	Localization-free beacon based routing protocol, route selection is based on multiple metrics (physical distance, link quality and residual energy).	Consider multiple metrics for next forwarder selection which create computational overhead. Moreover, physical distance calculation creates hello packet overhead.	Prolonged network lifetime, improved packet delivery ratio, reduced end-to-end delay and energy expenditure for both grid and random topologies.
CARP [19]	Distributed cross-layered routing protocol for multi-hop data delivery. Relay is selected on the basis of having a history of successful packet delivery to sink.	Use PING-PONG control packets for appropriate relay selection which is not efficient in relatively steady network. High mobility of sensor nodes will lead to accelerated hop-count growth.	High throughput, less energy consumption and reduced end-to-end delay.
E-CARP [20]	Distributed cross-layered reactive routing protocol for relatively steady network topology.	Reduced throughput and show high path loss due to mobility of sensor nodes	Prolonged network lifetime and reduce energy consumption when sensory data size is very large compared to control packet.
HydroCast [15]	Pressure based routing protocol for enhancing reliability and resolving void hole problem.	In HydroCast, the detour path may be invalid because of water current which increased its communication overhead as well as energy consumption.	High packet delivery ratio with limited co-channel interference.
FBR [23]	Location-aware routing protocol for networks containing both static and mobile nodes.	High energy dissipation along with the delay because of RTS/CTS.	Reduced unnecessary flooding.
DFR [25]	Location-aware directional flooding based routing protocol with controlled flooding technique to increase reliability.	Due to constant transmission power, more energy is utilized because more energy is used from source to destination. In sparse networks, eligible forwarder cannot be found when void hole occur.	High reliability with less communication overhead. Improved packet delivery ratio and less end-to-end delay.
VBF [27]	A geographic VBF routing protocol with a position based routing approach.	The static communication range leads to higher packet drop and low performance of the network. Thus, VBF is effective in dense deployment.	Achieved robustness, energy efficiency, and high data delivery ratio.
HHVBF [28]	A geographic VBF routing protocol with adaptive hop-by-hop location-based approach.	HH-VBF illustrate good behavior in even distribution network, however, when nodes deployment is uneven, the performance is greatly effected.	Improved the robustness of packet delivery in sparse networks with less energy consumption.
AHH-VBF [29]	A geographic VBF scheme that changes the pipeline radius dynamically for adjusting the forwarding region.	In AHHVBF, increasing the radius of the pipeline does not resolve the void hole problem.	Reduced end-to-end delay, energy consumption and improved data delivery ratio.

**Table 2:** Format of neighbor table.

**NeighID**	**NumNeighbor**	**HopSink**	**DistNeighbor**	**Depth**	**Timestamp**

**Table 3:** Parameter setting.

Parameter	Value
Maximum transmission power	50 W
Power threshold for receiving packets and idle state	158 mW
Maximum transmission range	2 km
Center frequency	12 kHz
Bandwidth	4 kHz
Data rate	32 kbps
Acoustic propagation speed	1.5 km/s
Deployment region	10 km × 10 km × 10 km
Number of sinks	9
Number of sensor nodes	100 to 500
Movement model	Random walk 2D mobility model
Header size	11 bytes
Payload	72 bytes
ACK or neighbor request	50 bits

**Table 4:** Performance evaluation parameters.

Metric	Definition
Packet delivery ratio (PDR)	PDR defines the success ratio of a network. It is computed using the number of packets successfully received at the destination over the total number of data packets generated from the network nodes.
Energy tax	Energy tax is measured in terms of power required to deliver a data packet from a source to the destination node. The unit of energy tax is joule (J).
End-to-end delay	The delay is defined based on the total time period required by a packet to reach the destination from the source. It includes propagation, transmission, holding time, receiving and processing delays. The unit of second is used to measure the delay.
Accumulative propagation distance (APD)	APD is the total distance required by a data packet to reach the destination successfully. It is measured in km.

**Table 5:** Performance trade-offs made by Re-Intar with WDFAD-DBR and Intar in terms of percentage. Positive sign shows that the performance of Re-Intar is improved over respective protocol and negative sign shows that the performance of Re-Intar is reduced.

Parameters		Number of Nodes								
		100	150	200	250	300	350	400	450	500
**WDFAD-DBR**	**PDR**	40.00%	31.08%	27.65%	18.05%	13.35%	9.46%	5.65%	4.06%	3.37%
	**Energy tax**	66.62%	34.44%	15.23%	9.66%	4.71%	2.42%	−0.057%	−1.45%	−1.63%
	**End-to-end delay**	38.20%	37.22%	37.85%	40.54%	37.82%	36.74%	36.78%	35.24%	34.13%
	**APD**	−22.27%	−14.48%	−11.09%	−6.46%	−5.64%	−4.85%	−3.43%	−3.38%	−3.87%
**Intar**	**PDR**	17.07%	15.67%	10.80%	7.16%	5.31%	3.84%	2.54%	1.71%	1.26%
	**Energy tax**	−2.81%	1.98%	5.44%	7.87%	9.36%	10.38%	11.98%	12.09%	12.55%
	**End-to-end delay**	−3.30%	3.23%	7.40%	10.30%	12.34%	13.79%	15.80%	15.98%	16.38%
	**APD**	−2.32%	2.46%	6.23%	8.88%	11.00%	12.12%	13.30%	13.90%	13.89%
